# Hand, Foot, and Mouth Disease Associated With Transient Hyperphosphatasemia

**DOI:** 10.7759/cureus.22066

**Published:** 2022-02-09

**Authors:** Anisha Verma, Benjamin Keaton, Aaron McGuffin

**Affiliations:** 1 Pediatrics, West Virginia School of Osteopathic Medicine, Lewisburg, USA; 2 Osteopathic Medicine, West Virginia School of Osteopathic Medicine, Lewisburg, USA

**Keywords:** hfmd, onychomadesis, alkaline phosphatase, enterovirus, coxsackievirus, petechiae, transient hyperphosphatasemia

## Abstract

A majority of pediatric outpatient practice involves managing familiar diseases that present in familiar ways. Occasionally, a familiar disease presents uniquely, which adds a diagnostic challenge and enhances the clinical experience of the clinician. We describe an 18-month-old male who presented to the clinic with a familiar disease but with unique additional findings. The patient had a one-day history of rash, subjective fever, and several episodes of non-bloody diarrhea. The rash included petechial lesions across his abdomen, groin, back, arms, and legs, as well as vesicular lesions in the mouth and on the palms and soles. A tentative diagnosis of hand, foot, and mouth disease (HFMD) was made. However, the presence of petechiae prompted further laboratory evaluation, including a complete blood count (CBC) and comprehensive metabolic panel (CMP). The CBC was unremarkable, but the CMP revealed an abnormally high serum alkaline phosphatase (ALP) level of 1,353 U/L (normal range: 53-128 U/L). The patient was subsequently diagnosed with an atypical presentation of HFMD associated with transient hyperphosphatasemia (TH). TH is characterized by a benign increase in serum alkaline phosphatase levels with an absence of liver or bone diseases. TH is usually clinically silent. Clinicians should consider the possibility of TH in pediatric patients who are found incidentally to have an elevated ALP, especially with a concomitant viral infection. An awareness and understanding of TH will prevent unnecessary additional testing and avoid undue parental anxiety.

## Introduction

The adage “common things are common” is often used to guide physicians when formulating a differential diagnosis for a patient, especially one whose clinical presentation may be atypical. In this case presentation, we present a “common” clinical diagnosis - hand, foot, and mouth disease (HFMD) - which had an uncommon presentation that included diffuse petechiae, elevated alkaline phosphatase (ALP), and a late finding of onychomadesis.

HFMD is a common clinical syndrome seen in the pediatric population, which is characterized by a wide variety of mucocutaneous and skin presentations including vesicles, macules, papules, and ulcers. The classical description and most common presentations are of a child younger than five years with vesicles in the mouth and on the hands and feet, hence the name. It is caused by enteroviruses, the most common culprit being coxsackievirus A16. Infection results from orally ingesting the virus after it is shed from the gastrointestinal or upper respiratory tracts. Outbreaks of HFMD often occur in daycares, schools, summer camps, and community centers. Cases are seen more frequently during the summer and fall seasons [1].

What made this case noteworthy was the widespread presence of petechiae, which prompted us to obtain laboratory work to investigate for other more serious diagnoses such as bacteremia, meningitis, immune thrombocytopenic purpura, thrombotic thrombocytopenic purpura, malignancy, hemolytic-uremic syndrome, and bleeding disorders. In the process of assessing for these conditions, a significantly elevated serum ALP level presented an additional clinical challenge for consideration.

## Case presentation

An 18-month-old male presented to the clinic with a one-day history of rash and several episodes of non-bloody diarrhea. His mother stated that the infant was irritable for two days prior to presentation and had several episodes where he “cried for hours” and was difficult to console. The initial rash was described as “small red dots,” which covered the abdomen, groin, back, arms, and legs. Vesicular lesions subsequently developed on the palms and soles and were also identified in the patient’s mouth. A tentative diagnosis of HFMD was made based on clinical presentation. The discomfort was relieved with ibuprofen, acetaminophen, sucking on frozen foods, and applying mineral oil to the affected areas.

The mother denied any history of cough, rhinorrhea, ear pain, tick exposure, new medications or medication changes, change in urinary volume, abdominal pain, muscle weakness, neck pain, weight loss, pallor, bleeding, bruising, or trauma. The patient was born at term without complications. His family history and the newborn screening tests were unremarkable. He had an inguinal hernia repair at two months of age. All immunizations were up to date.

**Figure 1:**
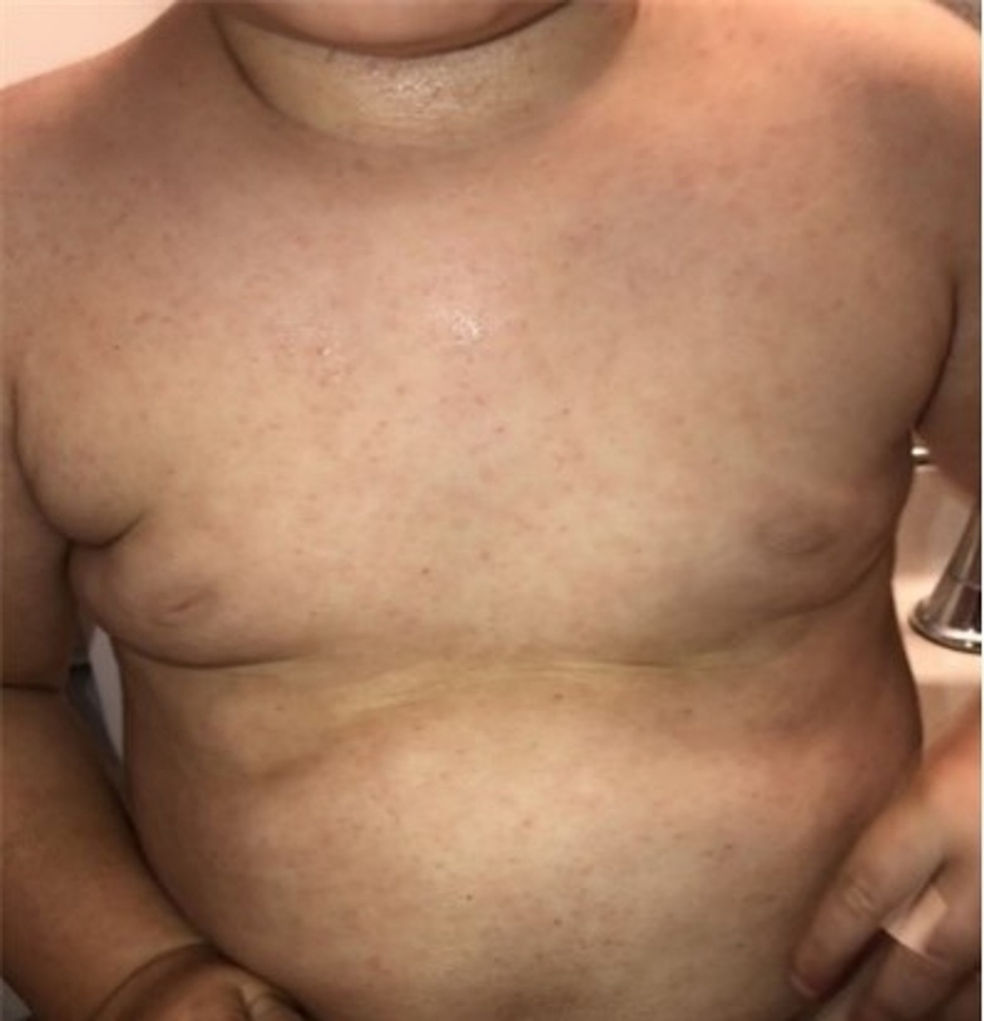
Petechial lesions spread across the abdomen

**Figure 2:**
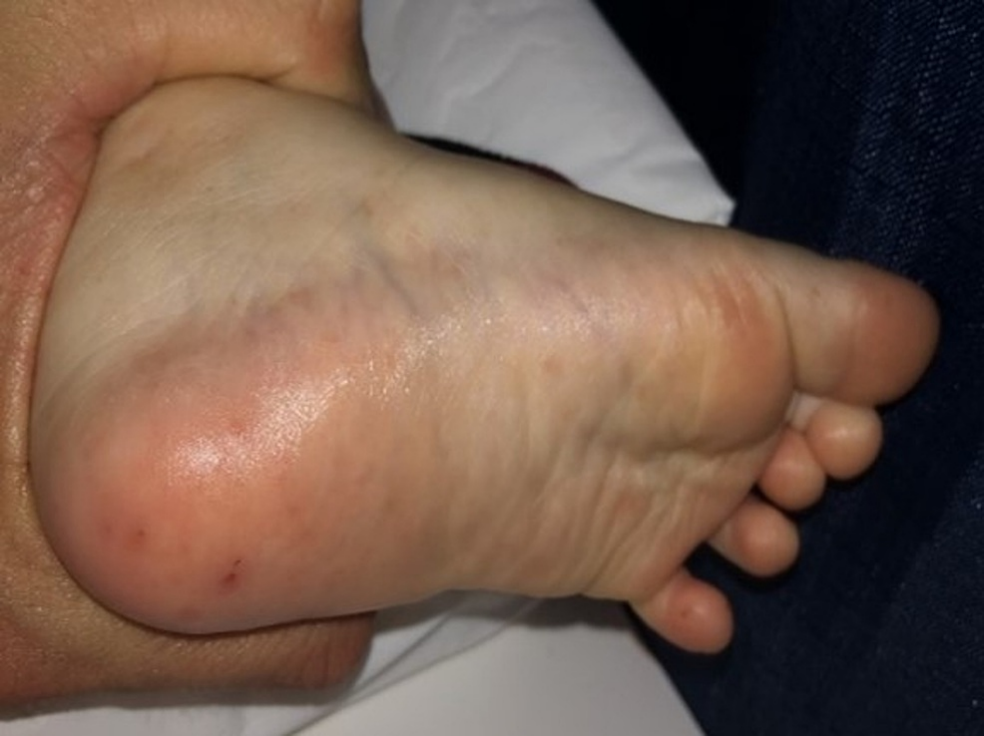
Purplish macular and vesicular lesions on the soles of the foot

**Figure 3:**
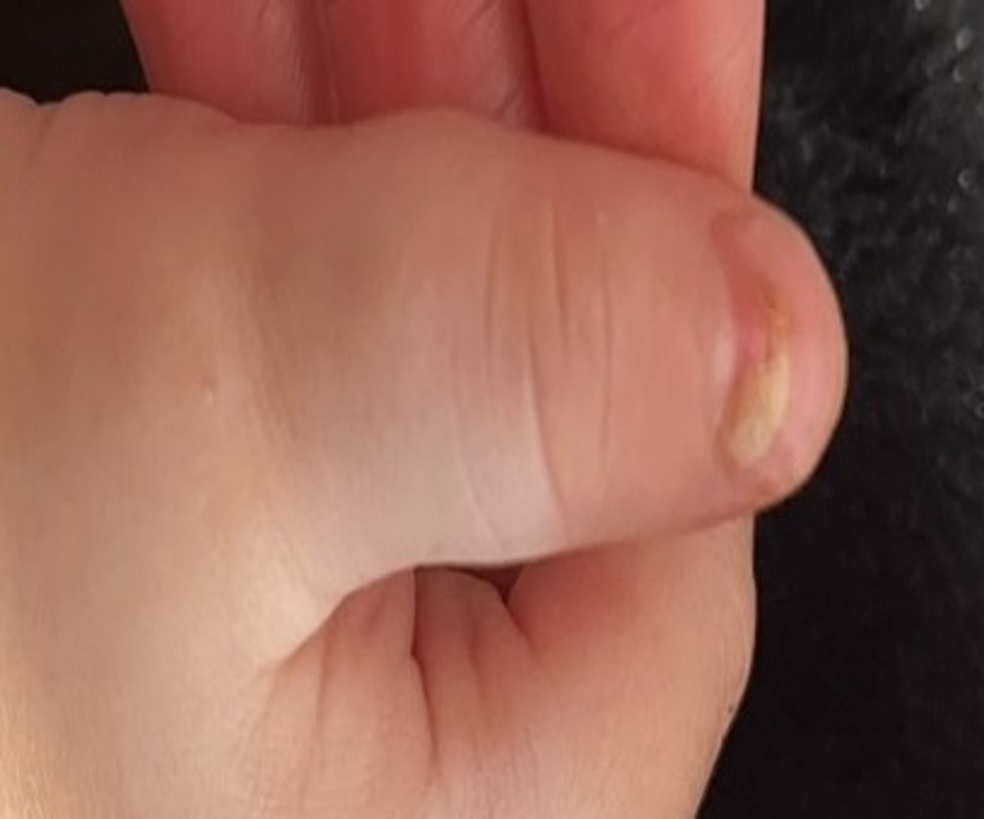
Onychomadesis of the nail bed

On examination, the recorded vital signs included a temperature of 97.2°F and a weight of 12.2 kg (86th percentile). The patient was ill-appearing and irritable. Examination of the head revealed no tenderness or swelling. The eyes revealed no redness or discharge, and the pupils were equal and reactive to light. The external ear and tympanic membranes were normal-appearing. The nose revealed no erythema or nasal discharge. The buccal mucosa revealed erythema and multiple vesicles. The throat had no exudate, erosions, or enlarged tonsils, and the uvula was midline. Dentition was normal for age. The neck was supple with some bilateral small cervical lymph nodes. The lungs were clear to auscultation bilaterally. The heart rate was normal without evidence of murmur, rub, or gallop. The abdomen was soft and non-tender. Table 1 lists the results of the complete blood count (CBC) and comprehensive metabolic panel (CMP). Petechiae were present on the arms, torso, back, groin, and legs (Figure 1). Additional pinpoint vesicles and purplish macules were noted on the hands and feet (Figure 2). His nails were normal-appearing during the initial visit but began to separate from their base approximately four weeks later (Figure 3).

**Table 1:** CBC and CMP results

Report	Result	Reference range	Units	
Albumin	4	3.3–5.5	g/dL	Normal
Alkaline phosphatase	1,353	53–128	U/L	High
ALT	24	10–47	U/L	Normal
AST	61	11–38	U/L	High
Calcium	10.2	8–10.3	mg/dL	Normal
Carbon dioxide	21	18–33	mmol/L	Normal
Chloride	106	98–108	mmol/L	Normal
Creatinine	0.4	0.6–1.2	mg/dL	Low
Glucose	92	73–118	mg/dL	Normal
Potassium	4.9	3.6–5.1	mmol/L	Normal
Sodium	142	128–145	mmol/L	Normal
Total bilirubin	0.6	0.2–1.6	mg/dL	Normal
Total protein	7.3	6.4–8.1	g/dL	Normal
Urea (BUN)	12	7–22	mg/dL	Normal
WBC	13.1	6–17	×10^3^/µL	Normal
RBC	4.63	3.8–5.2	×10^6^/µL	Normal
HB	12	11.3–14.1	×10 g/dL	Normal
HCT	35.7	31–41	%	Normal
MCV	77.1	70–90	fl	Normal
PLT	320	150–450	×10^4^/µL	Normal
Neutrophil	65.5	40–70	%	Normal
Lymphocyte	27.4	20–40	%	Normal
Other WBC	7.1	0–8	%	Normal

## Discussion

This patient presented with an atypical form of hand, foot, and mouth disease (HFMD) by virtue of petechiae. Atypical presentations of HFMD are more often seen in children less than three years of age and simply present with a more widespread, painless rash affecting the extremities, buttocks, genitals, and perioral region. It can also present as an eczema coxsackium eruption in areas with atopic dermatitis, a Gianotti-Crosti eruption (characterized by blisters on the skin that may or may not itch) that spares the torso, or a petechial or purpuric eruption as seen in our patient [2].

A normal white blood cell count and differential reassured that the patient does not have a bacterial infection. A normal platelet count was reassuring that the source of petechiae was not due to thrombocytopenia. Further, the blood cell count of the patient was reassuring that the patient was not experiencing an oncologic process.

At this point, the patient’s parents and clinician could be reassured that the petechiae are part of the suspected coxsackievirus presentation. However, what do we do about the elevated alkaline phosphatase (ALP)?

ALP is a family of isoenzymes that are found in several places in the body, including the kidney, bone, liver, placenta, gut, and white blood cells. Their primary function is to break down proteins and hydrolyze bone during the mineralization process. In children, the normal values for ALP are typically higher due to the rapid amount of bone development. However, ALP above the established age-based norm can be concerning for bone and hepatic pathologies. Bone pathologies include bone disorders such as rickets, healing fractures, Paget’s disease, and bone tumors. Hepatic pathologies include extrahepatic biliary obstruction, intrahepatic cholestasis, malignancy, and hepatitis [3].

As our patient did not have clinical findings suggestive of bone or hepatic pathologies, a diagnosis of transient hyperphosphatasemia (TH) was made. Transient hyperphosphatasemia (TH) is a benign condition characterized by an increase in serum ALP levels without the presence of liver or bone pathology. In TH, the specific isozyme of ALP that is elevated is distinct from the ALP isoenzymes that are commonly found. Sialylation of the ALP isoenzyme via sialic acid is known to sustain ALP levels in the serum and reduce its hepatic clearance, leading to a presentation of TH. This transient rise in ALP levels is mostly seen in young children between six and 24 months of age who are undergoing rapid osteoblastic activity [4].

Patients with TH do not exhibit clinical symptoms. The rise in ALP is usually an incidental finding recognized during laboratory testing for other reasons. TH is confirmed when ALP levels restore to normal range within four months. Liver or bone disorders must be reconsidered in patients who have sustained ALP levels beyond four months [4].

The exact cause of TH is unknown, and various factors have been suggested to have a role in its development, including viral infections in the late summer or early fall, as was the case with our patient. Temperature changes during the summer and fall may make respiratory infections more common, leading to an increase in TH cases [4].

The literature recognizes an association between TH and HFMD. Several publications report that recent viral infections involving *Enterovirus* species may predispose young children to develop TH [2,5,6]. To support this claim, various enterovirus antibodies have been found in the serum of patients diagnosed with TH, including echovirus 22, 3, 12, and 9, enterovirus 71, and coxsackie B4, B5, A16, B3, and A9, suggesting that these enteroviruses might indeed lead to TH in pediatric populations [5,6].

How certain viruses trigger this increase in serum ALP is unknown. However, one speculation is that mild liver injury induced by enterovirus infection may cause increased ALP serum levels in severe cases of HFMD [7]. In our case study, we add to the current findings suggesting that TH is associated with atypical HFMD.

## Conclusions

Our patient’s case serves as an important learning tool to recognize the potential atypical presentations of HFMD along with the appropriate management of elevated alkaline phosphatase if incidentally discovered.

Knowledge of this association should provide reassurance and obviate the need for further unnecessary workup in the absence of other compelling clinical findings that would prompt them otherwise. This will also help alleviate unnecessary parental anxiety, as they otherwise might be waiting for pending results indicating a serious medical condition.
